# A case of missed stab wound to the right heart diagnosed by cardiac imaging

**DOI:** 10.1093/ehjimp/qyaf078

**Published:** 2025-06-06

**Authors:** Ruchika Meel, Ricardo Gonçalves

**Affiliations:** Faculty of Health Sciences, Department of Internal Medicine, University of the Witwatersrand and Sandton Mediclinic, 7 York Road, Parktown. Johannesburg, 2193, South Africa; Glynnwood Hospital, Cardiology Practice, Benoni, South Africa

**Keywords:** stab chest, cardiac imaging, right heart

## Case

**Figure 1:**
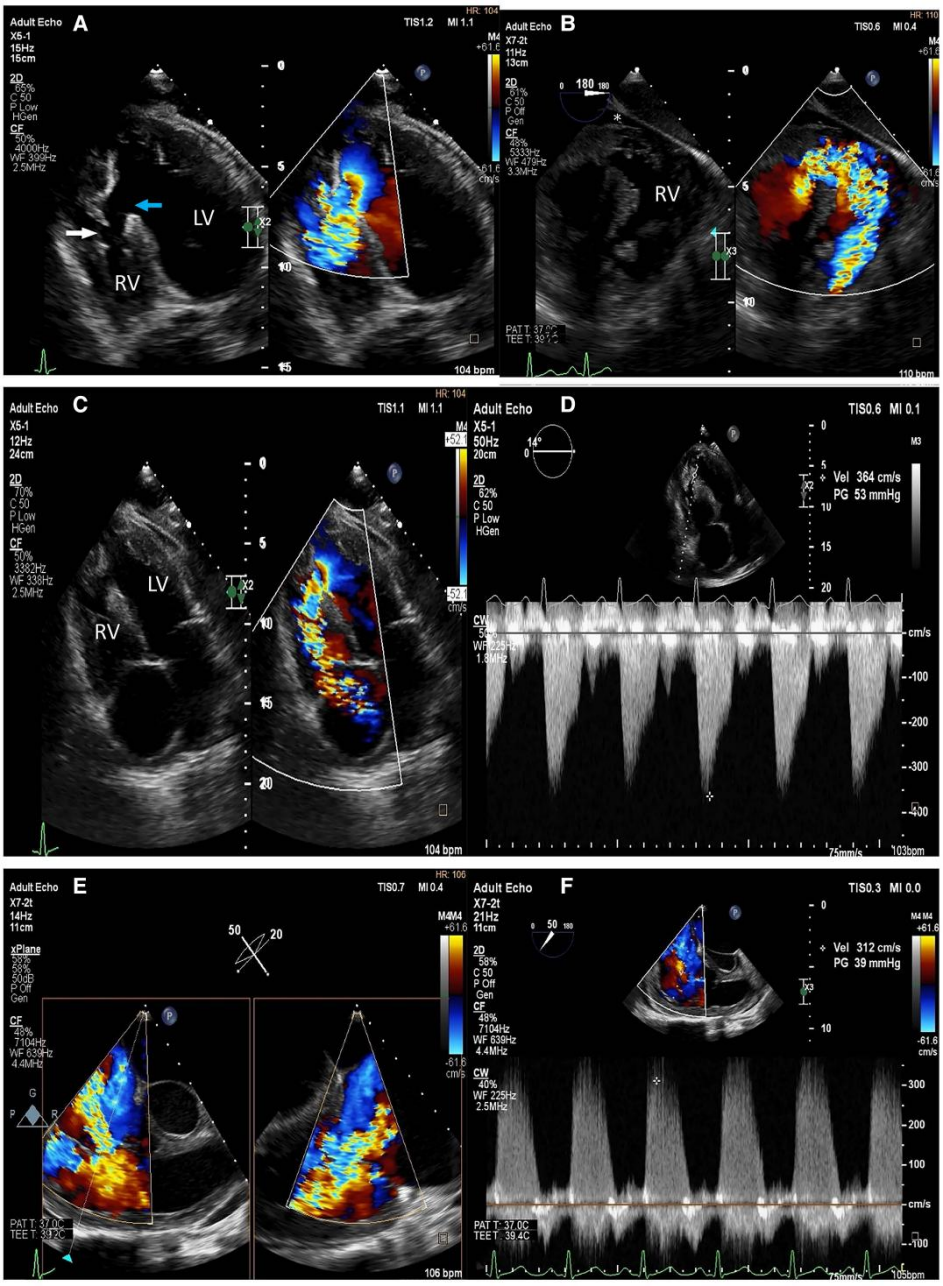
The transthoracic echocardiography (*A*), off-axis four-chamber(4C) view, shows (blue arrow) a large iatrogenic muscular ventricular septal defect (VSD) that measured 2 cm in size with a left to right shunt (LV, left ventricle; RV, right ventricle). There was also disruption of the moderator band (white arrow). (*B*) Shows the transoesophageal echocardiography transgastric view with a large serpentine jet extending form the defect and merging with the functional tricuspid regurgitation (TR) jet and a small pericardial effusion (marked with an asterisk). This can also be noted in the apical 4C colour compare images (*C*). (*D*) depicts the continuous wave (CW) Doppler with angle correction across the VSD and a peak gradient of 53 mmHg consistent with a large defect. (*E and F*) focuses on the TR jet with a right ventricular systolic pressure of 39 mmHg due to left to right shunting of blood.

A 20-year-old male presented with a history of stab wound to the chest. He had an intercostal drain inserted for haemothorax and was discharged. No cardiac imaging assessment was requested at the time of index presentation as cardiac injury was not suspected. He represented in predominant right heart failure a month later. On exam he had an audible tricuspid regurgitation murmur and elevated jugular venous pressure. Twelve lead electrocardiogram showed features of right ventricular strain and sinus tachycardia. Patient underwent transthoracic and transoesophageal echocardiography as depicted in *Figure 1* and *supplementary video*. A diagnosis of traumatic muscular ventricular septal defect (VSD) and moderator band injury was made. He was haemodynamically stabilized with medical therapy for heart failure while in hospital, and referred for urgent surgery. He underwent successful VSD patch closure, moderator band repair and tricuspid annuloplasty and continued to do well on follow-up.

This case highlights the importance of maintaining a high suspicion of myocardial injury in a case of stab chest. Alongside clinical examination, a detailed cardiac imaging exam must be undertaken to evaluate for all potential injuries. In this case in addition to the VSD, a crucial moderator band injury was noted on echocardiography, which was missed on initial clinical examination. Delayed diagnosis can result in complications such as heart failure, pulmonary hypertension due to left to right shunt as in this case. Further, moderator band injury can interfere with the right ventricle's electrical conduction system, possibly causing arrhythmias and impairing its pumping function. These complications increase the surgical risk.

## Supplementary Material

qyaf078_Supplementary_Data

## Supplementary data

**Consent:** Informed consent was obtained from the patient for the publication of this report.

**Funding:** None.

**Data availability:** No new data were generated or analysed in support of this research.

## Lead author biography

Prof. Ruchika Meel is a general cardiologist and clinician scientist in the Department of Internal Medicine at the University of the Witwatersrand, Johannesburg, South Africa. She is a Fellow of European Society of Cardiologyand an executive member of the Cardiac Imaging Society of South Africa. She has an interest in clinical cardiology and cardiovascular imaging. She has a passionate interest in research with to date 1 book publication, 4 book chapters, 49 research articles, and over 60 congress presentations. She is the recipient of Carnegie doctoral and post-doctoral awards. She is currently involved in research projects related to aortic aneurysms and valvular heart disease.

